# Cognitive behavioural therapy for the treatment of chronic fatigue syndrome in adults – a meta-analysis

**DOI:** 10.3389/fpsyt.2025.1647897

**Published:** 2025-10-31

**Authors:** Vivek Kolala, Billie La Rosa, Venkat Vangaveti, Kai Yang Chen

**Affiliations:** ^1^ Canberra Health Services, Canberra, ACT, Australia; ^2^ Serco, Townsville, QLD, Australia; ^3^ Queensland Health, Townsville, QLD, Australia; ^4^ James Cook University, Townsville, QLD, Australia

**Keywords:** cognitive behavioural therapy, chronic fatigue syndrome, meta-analysis, fatigue, physical functioning, myalgic encephalomyelitis, psychological treatment

## Abstract

The treatment efficacy of Cognitive Behavioural Therapy (CBT) for chronic fatigue syndrome (CFS) remains controversial. The purpose of this meta-analysis was to understand the short-term and long-term efficacy of CBT on different outcome measures on patients with CFS, as well as explore potential adverse effects. A meta-analysis was conducted in accordance with PRISMA 2020 guidelines (PROSPERO: CRD42023391926). PubMed, PsycINFO, CINAHL, SCOPUS, Web of Science, and EMBASE were searched from inception to September 2024 for randomised controlled trials (RCTs) of CBT in adults with CFS excluding Oxford-defined cohorts. Interventions included individual face-to-face, self-directed, and group CBT. The primary outcome was fatigue, with secondary outcomes of physical functioning, anxiety, depression, pain and quality of life. Risk of bias was assessed using the Cochrane Risk of Bias 2.0 tool. There were 12 studies included in this review, with 1799 total participants. All forms of CBT, when analysed together, was not found to have a statistically significant result (p = 0.12). Individual face-to-face CBT was found to have a large effect size in reducing fatigue (Cohen's d = 2.91, 95% CI 0.51 to 5.31, p=0.02). Self-directed CBT was found to have a large effect size in improving physical functioning (Cohen's d = -2.76, 95% CI -5.06 to -0.47, p=0.04). All other sub-analyses did not yield statistically significant results. There was inconsistent reporting of adverse effects, however no serious adverse effects were reported. High heterogeneity and incomplete reporting limit certainty. CBT as a treatment modality inherently leads to difficulties with blinding and bias. The results suggest that patients with milder disease may benefit more from self-directed CBT. Group CBT may not be as beneficial as other modalities. Guidelines provide mixed recommendations regarding CBT, therefore CBT may be offered as a supportive, non-curative option for adults with CFS. No funding was received for this review.

**Systematic review registration:**

https://www.crd.york.ac.uk/prospero/, identifier CRD42023391926.

## Introduction

Chronic fatigue syndrome (CFS) is a collection of symptoms characterised by persisting, debilitating fatigue. Patients with CFS have cognitive dysfunction, post-exertional malaise and unrefreshing sleep. Aetiology of CFS is unclear, with factors such as genetic predisposition, recent viral infections, and immune abnormalities thought to play a role.

CFS is thought to be an underdiagnosed condition, commonly affecting adults aged 40–60 years, and women more than men. The Centers for Disease Control and Prevention estimates CFS affects 17–24 million people worldwide (1). CFS imposes a significant economic burden globally, with studies suggesting annual costs approximately €40 billion across Europe, and US$36 to 51 billion in the United States (2, 3).

Current guidelines suggest that therapies such as Cognitive Behavioural Therapy (CBT) should be used for symptom reduction rather than curative treatment. The purpose of CBT in patients with CFS is to help establish stable routines, improve sleep habits, gradually increase activity levels, and address unhelpful patterns of thinking. Cognitive techniques are later introduced based on a fear-avoidance model. It is thought that maladaptive coping mechanisms, such as prolonged rest, avoidance of activity, or overexertion, are central in sustaining disability and fatigue (4).

There are several diagnostic criteria for diagnosing CFS, the most common being the Canadian Consensus Criteria 2003 (CCC), Centers for Disease Control and Prevention 1994 (CDC or Fukuda) criteria and the International Consensus Criteria 2011 (ICC) (5). Among the most debated diagnostic criteria is the Oxford criteria. It requires only the presence of medically unexplained fatigue lasting six months or longer, without requiring hallmark symptoms such as post-exertional malaise, non-restorative sleep, or cognitive impairment. As a result of this nonspecific criteria, studies that utilise the Oxford criteria may include patients with other general conditions. It has been argued that the population selected by the Oxford criteria may not be representative of patients diagnosed with more modern and stringent criteria (6). Several expert bodies have recommended that the Oxford criteria no longer be used in research or clinical practice (7, 8).

Until this study, all previous reviews have identified a statistically significant fatigue reduction in patients with CFS. Price et al.'s 2008 Cochrane review, and two meta-analyses published shortly after found CBT to have small to medium effect sizes in reducing fatigue (9–11). In 2020, a systematic review supported CBT use, however did not report an effect size, and also excluded studies with less than 45 participants (12). More recent reviews have also shown that CBT reduces fatigue, with a 2023 meta-analysis reporting a large effect size and a 2024 meta-analysis indicating a moderate effect (13, 14). The meta-analysis by Kuut et al. (13) only included randomised controlled trials (RCT) of a specific Dutch CBT protocol to reduce heterogeneity, which may not be applicable to investigate if CBT of a general approach is effective. Maas genannt Bermpohl et al., by contrast, included studies which determined patients who fulfilled the Oxford criteria for CFS, which may not be generalisable to the true CFS population. We are in need of an updated meta-analysis that examines whether a non-protocol based CBT is effective in a population with CFS that is not defined by the Oxford criteria (15, 16).

Patients with CFS also experience a range of secondary outcomes outside of fatigue. Pain, anxiety, depression, and reduced quality of life are reported at higher rates in this population compared to healthy controls (17).

This review seeks to determine whether adults with chronic fatigue syndrome diagnosed with officially recognised criteria, respond better to CBT compared to a non-CBT intervention. We hope to show that CBT does reduce fatigue severity both immediately following treatment, and at long-term follow-up. We hope to explore any potential adverse effects of CBT, as well as perform a meta-analysis of all RCTs available for CBT.

## Materials and methods

The databases PubMed, PsycINFO, CINAHL, SCOPUS, Web of Science and EMBASE were searched from inception till September 2024. The following MeSH terms and keywords, in varying combinations and forms, were used: 'chronic fatigue syndrome,' 'CFS,' 'myalgic encephalomyelitis,' 'systemic exertion intolerance disease,' 'post-viral fatigue syndrome,' 'chronic viral syndrome,' 'chronic fatigue immune deficiency syndrome,' AND 'cognitive behavioural therapy,' 'CBT,' 'cognitive-behavioural therapy,' and 'cognitive behavioural therapy.' We also hand-searched any citations and grey literature. We followed the Preferred Reporting Items for Systematic Reviews and Meta-Analyses (PRISMA) 2020 guidelines and registered our protocol in PROSPERO (registration number: CRD42023391926) on 15 January 2023 (18).

### Inclusion criteria

Articles were included if they were (1) RCTs that contained primary research data on the treatment of CFS and other synonymous conditions that met the diagnostic criteria of either CDC, ICC or equivalent, (2) used CBT as intervention and a non-CBT treatment comparator (including treatment as usual) was used, and (3) measured the change in level of fatigue pre and post-intervention.

### Exclusion criteria

Articles were excluded if (1) they exclusively used the Oxford criteria to determine participants of the study, (2) the subjects were non-humans, (3) the subjects were under the age of 18, and (4) they were not published in English and an English translation was not available. No restrictions were applied regarding inpatient or outpatient status, age, gender or comorbidities, to reflect the heterogeneity of routine clinical populations with CFS.

The adult population was specifically chosen for this study because disease trajectory and symptom profiles differ when compared to the paediatric population. Children and adolescents typically have shorter illness duration, lower rates of hallmark features of CFS, and fewer comorbidities. Recovery rates are also higher, suggesting that paediatric presentations may represent a more transient or evolving condition (19). This rationale parallels the exclusion of studies using the Oxford criteria, which similarly risks enrolling individuals with transient or non-specific fatigue (6).

### Data collection, synthesis, and article quality

Each article was screened independently by two reviewers, VK and BL. This was done by the abstract and title initially, then with the full text. Any disagreements were settled through discussion with the research group.

The primary outcomes measured were fatigue levels. Secondary outcomes gathered were changes to physical functioning, anxiety, depressive symptoms, quality of life, pain, long-term efficacy, alongside adverse outcomes of any intervention and comparator. Data was collected for pre-intervention and post-intervention. When follow-up data was available, the last data point post-intervention and duration of intervention were recorded. All outcome measures were transformed onto a standardised 0–100 scale to enable comparability across all studies. Scores were rescaled according to the published minimum and maximum values of each instrument. For example, a score of 28 on the Beck Depression Inventory (range 0 – 63) was rescaled to 44 on the 0–100 scale. Several types of CBT interventions were investigated, namely individual face-to-face, self-directed and group. Each study's results were pooled together according to these categories. When an RCT had multiple similar intervention arms, such as different types of CBT, the mean and standard deviations of these arms were combined. The same was done for comparator arms. Demographics of participants were also recorded if available, including biological gender, age, comorbidities, ethnicity, and country in which the study had taken place. Authors of RCTs with missing or unclear data were contacted for clarification.

Articles were assessed for methodological quality and risk of bias via the Cochrane Risk of Bias Tool version 2. A funnel plot and forest plot for the primary outcome measures were completed using SPSS (20). Meta-analysis using random-effects model was used to factor heterogeneity to pool data from studies. For sensitivity analyses, we explored the robustness of findings by conducting subgroup analyses according to CBT modality. Statistical heterogeneity was measured using the inconsistency I² test in which values greater than 50% were considered indicative of heterogeneity (21, 22).

## Results

### Included studies

**Figure 1:**
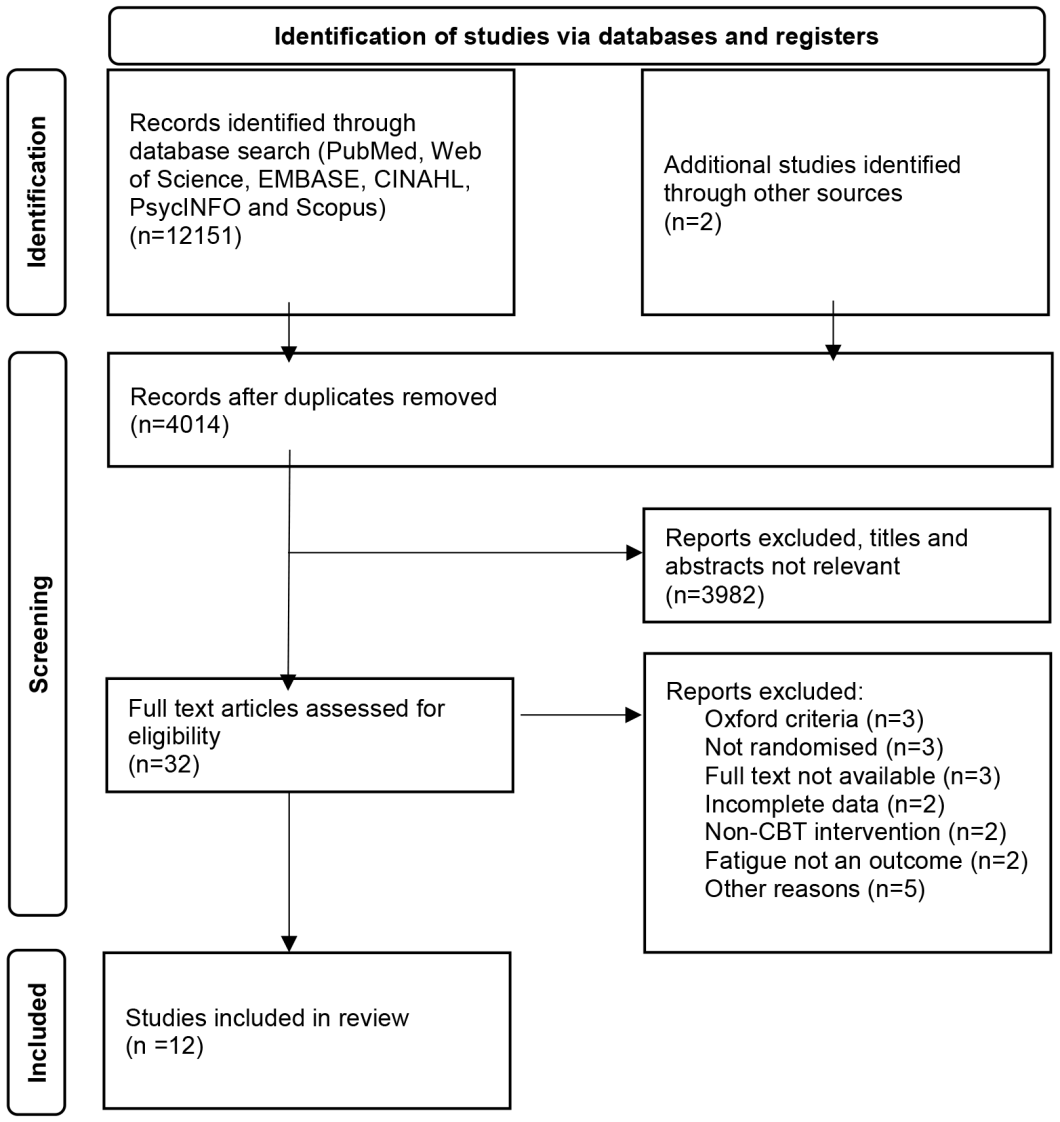
The preferred reporting Items for systematic reviews flowchart for the selection of studies.

In total, 12 studies were included in the final analysis (see **Figure 1**) (15, 16, 23–32). The total number of participants was 1799. The mean age of participants was 38 years, and 74% of participants in all included studies were female. Three studies commented on patient comorbidities (24, 26, 28), and one on whether patients underwent previous psychology (27) (see **Table 1** for Baseline characteristics of included studies). The average duration of illness for the individual face-to-face CBT group was 6.8 years, and 5.5 years for the self-directed CBT group. Of the included studies, six were conducted in the Netherlands, two in the United States of America, two in the United Kingdom, one in Norway, and one in Spain. Individual face-to-face CBT was used by five studies, with group CBT being used by four, and self-directed CBT used by three. The duration of the intervention ranged between three months and eight months. Frequency of the sessions were similar, the most frequent was held twice weekly, and the least was fortnightly (see Appendix 1). The baseline mean fatigue scores, with higher scores indicating worse fatigue, were 86.9/100 and 89.8/100 for individual face-to-face CBT and self-directed CBT respectively. The baseline mean physical functioning scores, with higher scores indicating higher functioning, were 49.2/100 and 54.9/100 for individual face-to-face CBT and self-directed CBT respectively. Authors of RCTs with missing data were contacted for additional information.

**Table 1:** Baseline characteristics.

Study	Total participants	Mean age		Female/male		Criteria fulfilled	Average duration of illness	Fatigue scale used	Country
*van der Schaaf* et al. (16)	51	32		51/0		CDC	5.2 years	CIS	Netherlands
*Gotaas* et al. (15)	236	35		149/87		CDC, CCC in last year of inclusion	4.8 years	Chalder Fatigue Scale	Norway
*Janse* et al. (29).	240	37		145/95		CDC	5 years	CIS	Netherlands
*Wiborg* et al. (23)	204	38		157/47		CDC	9.1 years	CIS	Netherlands
*Tummers* et al. (30)	123	36		96/27		CDC	4.5 years	CIS	Netherlands
*Núñez* et al. (24)	120	43		108/12		CDC	2.7 years	HAQ weakness score	Spain
*Lopez* et al. (31)	69	46		61/8		CDC	Not reported	Profile of Mood State Fatigue	United States of America
*Knoop* et al. (33)	169	38		134/35		CDC	7 years	CIS	Netherlands
*Jason* et al. (26)	114	44		95/19		CDC	7.4 years	Fatigue Severity Scale	United States of America
*O'Dowd* et al. (27)	153	41		102/51		CDC	50% of patients had symptoms over 5 years	Chalder Fatigue Scale	United Kingdom
*Prins* et al. (32)	278	37		197/81		CDC, apart from 4 out of 8 exclusion criteria being required	5.6 years	CIS	Netherlands
*Deale* et al. (28)	60	34		41/19		CDC and Oxford	4 years	Fatigue problem rating and fatigue questionnaire	United Kingdom

The most common fatigue rating scale was the Checklist Individual Strength (CIS), used by six studies, followed by the Chalder Fatigue Scale, used by two studies. Other fatigue scores used by included articles were Health Assessment Questionnaire (HAQ) weakness score, Profile of Mood States (POMS) Fatigue Score, Fatigue Severity Scale, Fatigue Problem Rating, and Fatigue Questionnaire. Secondary outcomes measured were physical functioning, quality of life, pain, depression, and anxiety (see **Table 1**).

### Excluded studies

There were two studies excluded as CBT was not compared to another intervention (34, 35). Other reasons for exclusion were if fatigue was not an outcome measure (36, 37), unclear inclusion criteria (38) or unclear fatigue scoring values (39).

### Fatigue

**Figure 2:**
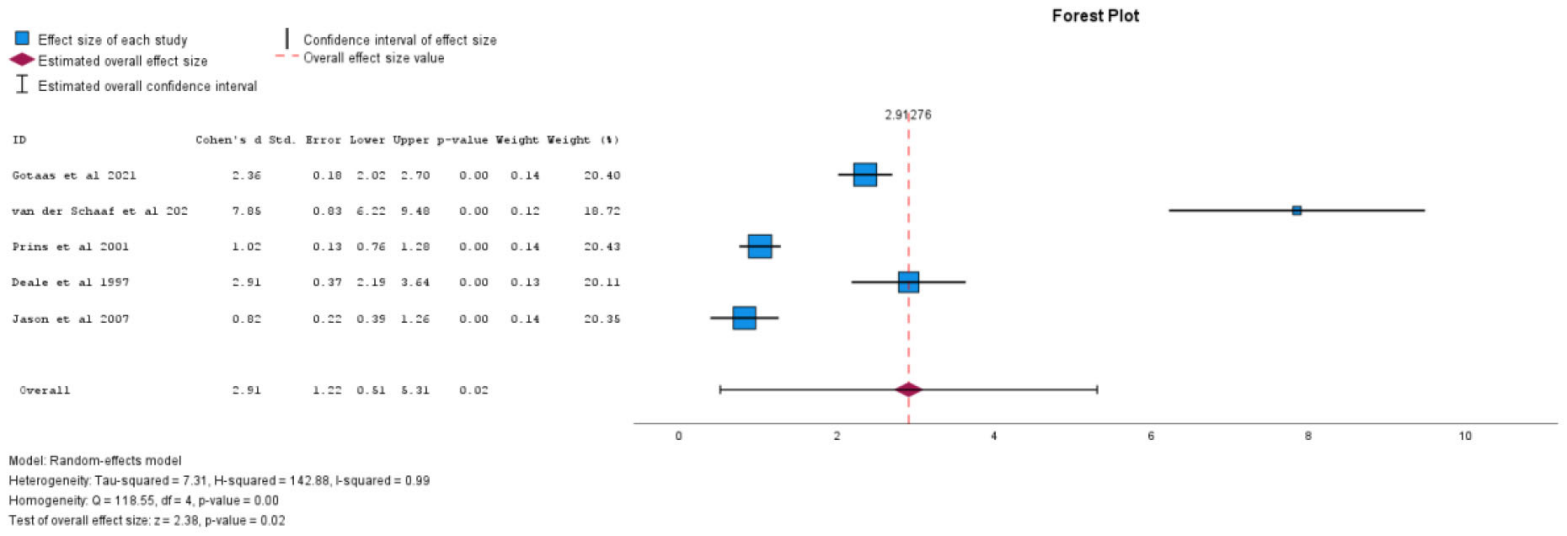
Individual face-to-face CBT vs comparator for effect on fatigue levels (positive values equal decreasing fatigue).

There were 12 studies that contributed to the primary outcome meta-analysis. Significance in subgroup (moderator) analyses was determined by p < 0.05 for the pooled effect within each subgroup, using random-effects models. For fatigue, individual face-to-face CBT showed a significant effect (Cohen’s d = 2.91, 95% CI 0.51 to 5.31, p = 0.02) as shown in **Figure 2**, while group (Cohen's d = -0.59, 95% CI -14.93 to 13.75, p = 0.88) and self-directed CBT (Cohen's d = 0.96, 95% CI -0.23 to 2.16, p = 0.07) were non-significant (40). All forms of CBT, when analysed together, was not found to have a statistically significant result (Cohen's d = 1.49, 95% CI -0.37 to 3.36, p=0.12). CBT efficacy at six months post-treatment (Cohen's d = 2.26, 95% CI -0.41 to 4.94, p = 0.07) also did not yield statistically significant results in reducing fatigue. See **Table 2** for sub-analyses.

**Table 2:** Sub-analyses.

Outcome	Intervention (CBT)	Studies	Cohen's d (95% CI)	P value	I^2^
*Fatigue (positive value equals reduced fatigue)*	All forms	van der Schaaf et al, Gotaas et al, Janse et al, Wiborg et al, Tummers et al, Núñez et al, Lopez et al, Knoop et al, Jason et al, O'Dowd et al, Prins et al, Deale et al	1.49 (-0.37 to 3.36)	p = 0.12	97%
	Individual face-to-face	van der Schaaf et al, Gotaas et al, Jason et al, Prins et al, Deale et al.	2.91 (0.51 to 5.31)	p = 0.02	99%
	Group	Wiborg et al, Núñez et al, Lopez et al, O'Dowd et al	-0.59 (-14.93 to 13.75)	p = 0.88	96%
	Self-directed	Janse et al, Tummers et al, Knoop et al	0.96 (-0.23 to 2.16)	p = 0.07	88%
	6-month follow-up	O'Dowd et al, Prins et al, Deale et al	2.26 (-0.41 to 4.94)	p = 0.07	95%
*Physical functioning (negative value equals improved physical functioning)*	All forms	Gotaas et al, Janse et al, Wiborg et al, Tummers et al, Núñez et al, Knoop et al, Jason et al, O'Dowd et al, Prins et al, Deale et al	-2.05 (-5.71 to 1.61)	p = 0.24	100%
	Individual face-to-face	Gotaas et al, Jason et al, Prins et al, Deale et al	-4.48 (-8.93 to -0.02)	p = 0.05	99%
	Group	Wiborg et al, Núñez et al, O'Dowd et al	1.98 (-18.63 to 22.59)	p = 0.72	100%
	Self-directed	Janse et al, Tummers et al, Knoop et al	-2.76 (-5.06 to -0.47)	P = 0.04	94%
	6-month follow-up	O'Dowd et al, Prins et al, Deale et al	-2.81 (-11.2 to 5.59)	p = 0.29	99%
*Anxiety (negative value equals increased anxiety)*	All forms	Jason et al, O'Dowd et al	12.18 (-154.05 to 178.41)	p = 0.52	100%
*Depression (negative value equals increased depressive symptoms)*	All forms	Jason et al, O'Dowd et al, Deale et al	-0.28 (-23.28 to 22.72)	p = 0.98	93%
*Pain (negative value equals increased pain)*	All forms	van der Schaaf et al, Núñez et al, Jason et al	4.32 (0.09 to 8.55)	p = 0.05	83%
*Quality of life (negative value equals improved quality of life)*	All forms	Jason et al, Prins et al, Lopez et al.	-3.11 (-13.6 to 7.39)	p = 0.33	100%

### Physical functioning

**Figure 3:**
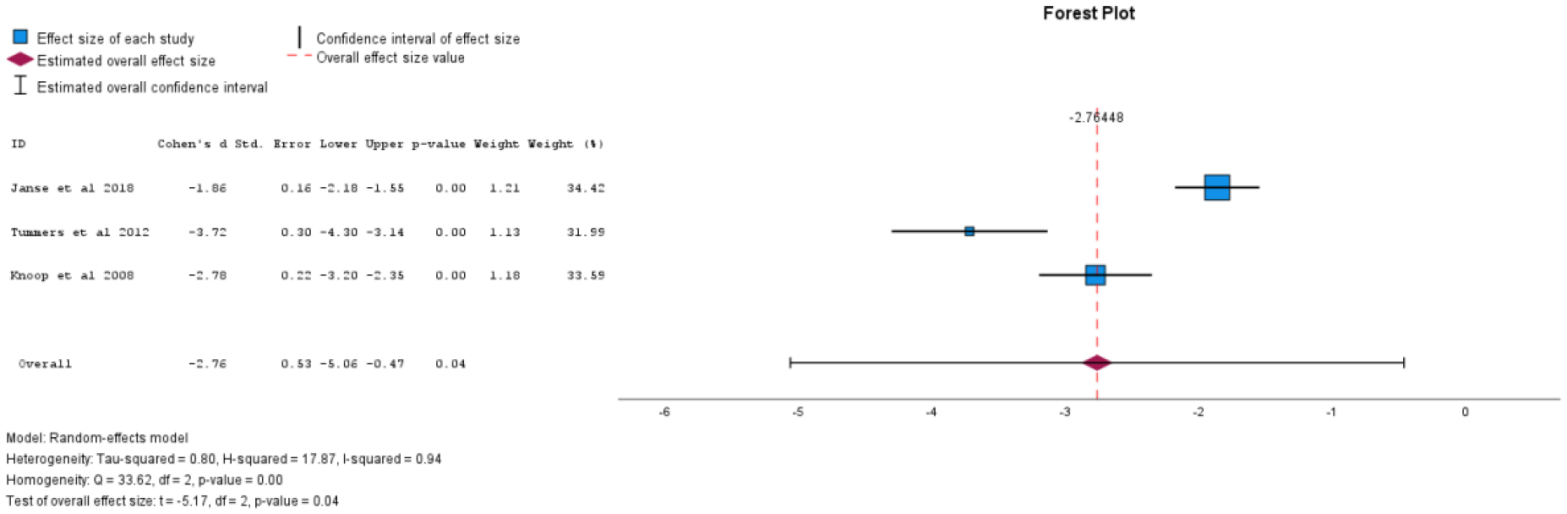
Self-directed CBT vs comparator for effect on physical functioning (negative value equals improving physical functioning).

There were ten studies included in the analysis of physical functioning. Self-directed CBT was found to have a large effect size and be statistically significant in improving physical functioning (Cohen's *d =* -2.76, 95% CI -5.06 to -0.47, p = 0.04), as shown in **Figure 3**. Individual face-to-face CBT was not found to be statistically significant in improving physical functioning (Cohen's *d* = -4.48, 95% CI -8.93 to -0.02, p = 0.05). Sub-analyses for all forms of CBT when analysed together(Cohen's d = -2.05, 95% CI -5.71 to 1.61, p = 0.24), group CBT (Cohen's d = 1.98, 95% CI -18.63 to 22.59, p = 0.72), and CBT efficacy at six months post-treatment (Cohen's d = -2.81, 95% CI -11.2 to 5.59, p=0.29) did not find statistically significant results. See **Table 2** for sub-analyses.

### Other secondary outcomes

There were three studies that measured depression, quality of life pain, while anxiety was measured by two studies. Analysis for anxiety (Cohen's d = 12.18, 95% CI -154.05 to 178.41, p = 0.52), depression (Cohen's d = -0.28, 95% CI -23.28 to 22.72, p = 0.98), pain (Cohen's d = 4.32, 95% CI 0.09 to 8.55, p = 0.05) and quality of life (Cohen's d = -3.11, 95% CI -13.6 to 7.39, p = 0.33) did not find statistically significant results (See **Table 2**).

### Small-study effects and sensitivity analysis

A funnel plot for the primary outcome (fatigue) showed no clear evidence of small-study effects, such as publication bias, and Egger's test was not significant p = 0.85. Sensitivity analyses were performed via subgroup analyses by CBT modality (**Table 2**), confirming that individual face-to-face CBT significantly reduced fatigue and self-directed CBT improved physical functioning. High heterogeneity (I² > 90%) suggests substantial variability due to differences in intervention protocols, participant characteristics, or study design, but modality-specific effects remained robust.

### Adverse events and dropout rates

Three studies reported adverse events in the intervention groups and four for the comparator groups. Núñez et al. found the intervention group, who were treated with group CBT, to have poorer scores for physical functioning and bodily pain than the comparator group (24). Janse et al. reported that approximately 15% of the intervention group and 26% of the control group reported adverse effects of increased fatigue, pain and or distress (29). Gotaas et al. noted that 2% in the intervention group and 5% in the comparator complained of increased fatigue, nausea and pain (15). Jason et al. found the comparator group to experience negative pain and memory changes (26). No study reported serious adverse events.

All included studies reported on the number of dropouts, withdrawals from the study, or the number lost to follow-up. Dropout rates varied from 8% to 43.4%. The average dropout rate for the intervention group was 18.8% and 12.5% for the comparator group. Reasons for dropout included distance to treatment, monetary strains and lack of motivation (15). See Appendix 1 for further details.

### Quality analysis

Most studies had some concerns in terms of overall risk of bias, with two studies at high risk. There were participants who were later found to have a medical explanation for fatigue during the studies, and those who received additional treatment outside of the intended intervention were included. No studies enforced adherence to CBT alone, and therefore were deemed to have some concerns in one component of the risk analysis. A recent study was categorised as having some concerns for bias in the randomisation process as all patients were female, and because of missing data. The study's primary outcome was to assess neural changes with CBT rather than CBT's efficacy on fatigue, hence the methodology was structured around this goal. All but one study were deemed low risk of bias in reporting results as multiple outcome measurements were reported, and data was produced in accordance with a pre-specified analysis plan. See **Table 3** for quality analysis details.

**Table 3:** Quality analysis.

Study	Risk of bias from randomisation process	Risk of bias due to deviations from the intended interventions (effect of assignment to intervention)	Risk of bias due to deviations from the intended interventions (effect of adhering to intervention)	Risk of bias due to missing outcome data	Risk of bias in measurement of outcome	Risk of bias in selection of reported result	Overall risk of bias
*van der Schaaf* et al. (16)	Some concerns	Low risk	Some concerns	Some concerns	Low risk	Low risk	Some concerns
*Gotaas* et al. (15)	Low risk	Low risk	Some concerns	Low risk	Low risk	Low risk	Some concerns
*Janse* et al. (29)	Low risk	Low risk	Some concerns	Low risk	Low risk	Low risk	Some concerns
*Wiborg* et al. (23)	Low risk	Low risk	High risk	Low risk	Low risk	Low risk	High risk
*Tummers* et al. (30)	Low risk	High risk	Some concerns	Low risk	Low risk	Low risk	High risk
*Núñez* et al. (24)	Low risk	Low risk	Some concerns	Low risk	Low risk	Low risk	Some concerns
*Lopez* et al. (31)	Some concerns	Some concerns	Some concerns	Low risk	Low risk	Low risk	Some concerns
*Knoop* et al. (33)	Low risk	Low risk	Some concerns	Low risk	Low risk	Low risk	Some concerns
*Jason* et al. (26)	Low risk	Low risk	Some concerns	Some concerns	Low risk	Low risk	Some concerns
*O'Dowd* et al. (27)	Low risk	Low risk	Some concerns	Low risk	Low risk	Low risk	Some concerns
*Prins* et al. (32)	Some concerns	Some concerns	Some concerns	Low risk	Low risk	Some concerns	Some concerns
*Deale* et al. (28)	Low risk	Low risk	Some concerns	Low risk	Low risk	Low risk	Some concerns

## Discussion

### Strengths

One major strength of our meta-analysis is that we excluded RCTs that used the Oxford criteria. Use of the Oxford criteria can lead to inclusion of other fatigue-related conditions, and possible self-resolving fatigue. Such factors may have accounted for the increased efficacy of CBT in other reviews that used the Oxford criteria (41). By restricting this analysis to studies using more specific case definitions, the results are more likely to reflect outcomes in patients with CFS consistent with current diagnostic frameworks. In contrast, inclusion of Oxford-defined CFS patients risks reduced generalisability to true CFS cohorts. This approach contrasts with recent reviews such as that of Maas genannt Bermpohl et al., which included Oxford-defined cohorts, and therefore may have drawn conclusions less representative of the true clinical CFS population (14).

This study investigated long-term efficacy of CBT for CFS, particularly after the cessation of treatment. There were no benefits sustained in the long-term following CBT. CBT's therapeutic benefits may wane over time. Changes in life circumstances or the addition of new stressors may necessitate reinforcement of previously learnt techniques (42). Support for the need for reinforcement sessions comes from other conditions, such as Obsessive-Compulsive Disorder, where patients are at a lower risk of relapse if they receive booster sessions compared to those who do not (43).

This study also compared the efficacy of different methods of delivery of CBT, which had not been investigated previously. We did not include a size restriction in the exclusion criteria, with the intention to capture smaller RCTs, however no additional RCTs were found as compared to other studies that excluded smaller RCTs.

### Limitations

A limitation of this study comes from the nature of psychological therapies. It is difficult to blind the participants from the type of therapy received. It is also not possible to blind the therapist from the type of treatment provided. This inherently may lead to performance bias from the participants, and alter their reported outcomes (44). We also noted that many of the RCTs did not specify which participants were utilising pharmacotherapy in addition to CBT. As a result, there were no studies deemed as low risk of bias in our quality analysis.

We did not restrict studies to those employing a specific CBT protocol or manual. This resulted in a diverse range of RCTs, where techniques utilised, length of sessions, and duration between sessions were not standardised. Other confounding factors that may have affected outcomes were comorbid mental illnesses, which was reported by three studies, and past exposure to psychological interventions, which was reported by one study. The presence of comorbid psychiatric conditions may influence illness perceptions and coping styles that shape treatment response (45). While some studies have associated comorbidities with a poorer quality of life, they do not necessarily exacerbate symptom severity (46). With respect to prior psychotherapy, research has shown that patients who have engaged in previous psychotherapy can have a higher level of self-efficacy, consequently improving receptiveness and response to psychology (47).

The average dropout rate in the intervention groups was 18.8%. A meta-analysis of dropout rates of CBT for various psychological disorders found a rate of 26.2%, with outpatient settings and e-therapy having higher attrition rates (48). Other common reasons as suggested in literature for dropout from CBT can include a mismatch between patient expectations and style of therapy, perceived lack of improvement, and overwhelming feelings due to confronting distressing thoughts (49–52). Our study supports that the dropout rate for CBT used for CFS may be similar to CBT used for other psychological disorders.

While some trials provided quantitative data on adverse events, they were generally documented inconsistently and often lacked standardised definitions or information on severity. This is not uncommon for research in psychotherapy, which often have inconsistent reporting of adverse events (53). Although no serious adverse events were reported, caution is warranted when interpreting these findings.

We were unable to conduct meta-regression analyses of potential moderators such as sex distribution, treatment duration, or number of CBT sessions due to incomplete and inconsistent reporting across studies.

### Results and comparison to current literature

We found that CBT in all forms of delivery, was not statistically significant in reducing fatigue. Up until this study, all previous reviews have identified a statistically significant fatigue reduction in patients with CFS, including two which had been published since completing our database search. The Cochrane review by Price et al. (9) found CBT to have a medium effect size, with a Cohen's *d* of 0.39 against usual care and 0.43 against other psychological therapies in reducing fatigue. Shortly after, in 2008 and 2011, two meta-analyses were published which had results aligning with Price et al. (10, 11). More recently, a 2023 meta-analysis found that CBT significantly reduced fatigue severity (β = −11.46, 95% CI −15.13 to −7.79, p < 0.001) (13). A 2024 meta-analysis yielded similar results (g = -0.52, 95% CI -0.69 to -0.35) (14).

We did not find CBT in all forms of delivery to be statistically significant in improving physical functioning. Physical functioning was found to have improved in all of the aforementioned reviews which investigated this, apart from Price et al. (9). One potential reason for this is differences in the eligible population, since both our review and Price et al. (9) excluded adolescent RCTs, whereas Castell et al. (11), Malouff et al. (10) and Kuut et al. (13) included them. Maas genannt Bermpohl et al. (14) applied the same adult-only inclusion criteria, but broadened physical functioning to also include disability and health status. Kuut et al. (13) in comparison, restricted their analysis to the SF-36 Physical Functioning scale, which may have increased consistency and likelihood of detecting an effect.

Depression improved in two out of three previous reviews that investigated mood outcomes (11, 14), whereas anxiety improved in all three, including Price et al. (9). In contrast, our review did not find a statistically significant result in either domain. The number of RCTs contributing to each analysis may partly explain these differences, as Castell et al. (11) and Maas genannt Bermpohl et al. (14) utilised eight or more RCTs for each analysis, whereas Price et al. (9) and our review utilised four or fewer. This, however does not explain why Price et al. (9) still found a small but significant improvement in anxiety whereas we did not. Potentially the inclusion of RCTs with Oxford-defined populations accounted for this.

Pain was not investigated in any previous meta-analyses. Quality of life was not found to be significant in one study which investigated it as an individual outcome (9). We did not find statistically significant effects for either secondary outcome. Pain is a complex, multifactorial symptom that may require multimodal approaches beyond CBT (54). Similarly, quality of life is a broad, multidimensional construct and may be less amenable to change from CBT alone, particularly if other symptoms remain unaddressed (55).

CBT efficacy for reducing fatigue levels at 6 months post-treatment did not reach statistical significance. Two previous reviews found conflicting results to ours, however this could be due to the inclusion of RCTs with Oxford-defined populations or adolescents (10, 14). By comparison, Price et al. (9) observed a small reduction in mean fatigue severity at follow-up that did not translate into higher clinical response rates, suggesting limited clinical significance. We did not find CBT efficacy for improving physical functioning levels at 6 months post-treatment to be statistically significant. No other reviews reported follow-up benefit for this either.

The primary reasons for the difference in included RCTs between our review and that of others are due to conditions regarding the CFS diagnostic scale, CBT protocol, and RCTs involving adolescents. Our study, unlike other reviews, did not restrict to a specific protocol or fatigue scale, which allowed for a wide range of studies from multiple authors and countries. The Dutch CBT protocol examined in the meta-analysis by Kuut et al. is highly structured, explicitly sets recovery as a treatment goal, and incorporates prescriptive activity scheduling and strategies to shift attention away from symptoms (13, 56). In contrast, our meta-analysis included a broader mix of protocols, which differ in their structure, activity planning, and therapeutic focus. While this increased generalisability across settings, it also introduced greater heterogeneity, therefore results should be interpreted with appropriate caution.

The inclusion of adolescents also may have accounted for the positive results found by other reviews. Research has shown that younger patients with CFS, particularly adolescents, benefit the most from CBT (13). This is due to their increased cognitive flexibility and greater resilience in their hypothalamic-pituitary-adrenal axis function which leads to improved adaptability to treatments (57).

To date, no other meta-analysis has explicitly compared the efficacy of different CBT modalities for chronic fatigue syndrome. Individual face-to-face CBT was found to be effective in reducing fatigue in our review, but not physical functioning. On the other hand, self-directed CBT was found to show a statistically significant result in physical function improvement but not for fatigue.

A benefit of self-directed CBT is that patients can work through the modules at their own pace. CBT has been criticised as although it aims to address thoughts and beliefs about CFS which impair recovery, it can also lead to overexertion, in particular when combined with graded exercise therapy (58). Self-directed CBT may minimise this risk, as patients have more autonomy over their activities, and therefore lead to an improved physical functioning score. In contrast, individual face-to-face CBT may place more emphasis on challenging unhelpful cognitions, leading to an improvement in fatigue scores, which are more subjective than physical functioning scales (59).

Personalisation of CBT treatment for CFS is a recommendation made by some sources (60), however the results of self-directed CBT challenge this as the modules were standardised. Although all three self-directed CBT RCTs included access to a therapist, Janse et al. did not find a significant correlation between therapist access and efficacy, as they had two intervention arms with differing clinician input (29). The mean baseline physical functioning was higher in the self-directed CBT group compared to individual face-to-face CBT group (54.9/100 compared to 49.2/100). This could suggest that self-directed CBT may be more viable for patients with a milder disease.

Our findings are in agreement with the European Network on Myalgic Encephalomyelitis/Chronic Fatigue Syndrome (EUROMENE) guidelines. They state that CBT should be used as a supportive rather than a curative treatment. These guidelines however highlighted concerns regarding the quality of some studies, and the uncertainty regarding adverse effects (61). In contrast, both ME/CFS Clinician Coalition and the US Centers for Disease Control and Prevention, have criticised the use of CBT and have removed it as a recommendation for CFS treatment (62). They highlight that CBT does not treat the multisystem biological impairment, and how some current studies have flawed methodology with the use of broad diagnostic criteria with inadequate follow-up of adverse events.

### Practice recommendations

Overall, our meta-analysis suggests that individual face-to-face CBT and self-directed CBT have a role in symptom management of CFS. For patients with milder symptoms, self-directed CBT should be utilised. At this stage group CBT may not be as beneficial as the other modalities.

From our review, secondary outcomes of depression, anxiety, pain and quality of life did not improve with CBT in patients with CFS. These limitations need to be kept in consideration when offering this treatment to patients.

Clinicians prescribing CBT for treatment should be aware of dropout rates for CBT. Both health systems and patients incur high costs to engage in CBT, such as from training CBT workforce, providing specialised settings, and other administrative expenses. The lack of long-term efficacy of CBT shown in this review further compounds this, as booster sessions may be required. Health services should consider these issues and their potential financial implications.

### Further research recommendations

The longitudinal efficacy of CBT is a domain where more research is required. From our study, there was some suggestion that the positive benefits continue after treatment, but we have not been able to ascertain for how long this effect continues. Research on the efficacy of booster sessions would guide management plans and when patients are recommended to revisit CBT. Additionally, future trials should continue to report adverse effects for a clear understanding of the overall risk-benefit of this intervention.

We noted that fatigue and physical functioning were impacted differently depending on the modality of CBT. RCTs exploring this dynamic would be beneficial in understanding what components of the therapy are more effective for each domain. Further research to explore CBT's efficacy on secondary outcomes such as depression, anxiety, pain and quality of life would also assist with understanding the capabilities of this treatment.

In our analysis, we did not explore the use of CBT with other interventions, for example augmentation with pharmacotherapy. Further studies investigating which treatments work synergistically with CBT can help form management plans. Research on reasons for dropout would also assist in forming management plans, as these factors can be targeted to improve adherence.

Greater consistency in reporting participant and treatment characteristics in future trials would help enable meta-regression analyses to explore whether factors such as sex distribution, treatment duration, or number of CBT sessions influence outcomes.

## Conclusion

This meta-analysis found efficacy of individual face-to-face CBT in reducing fatigue, and self-directed CBT in improving physical functioning. The latter may be suitable for patients with milder forms of the illness. Interpretation should be cautious given the high heterogeneity (I² > 90%) across studies. No serious adverse effects were found, although adverse effects were variably documented. Further research into the utility of self-directed CBT is recommended, alongside strategies to improve adherence, long-term efficacy, and combination treatments.

## Data Availability

The original contributions presented in the study are included in the article/**Supplementary Material**. Further inquiries can be directed to the corresponding author.

## References

[1] CDC . Epidemiology (2021). Available online at: https://www.cdc.gov/me-cfs/healthcare-providers/presentation-clinical-course/epidemiology.html (Accessed October 10, 2022).

[2] Pheby DFH Araja D Berkis U Brenna E Cullinan J de Korwin JD . The development of a consistent europe-wide approach to investigating the economic impact of myalgic encephalomyelitis (ME/CFS): A report from the european network on ME/CFS (EUROMENE). Healthcare (Basel). (2020) 8. doi: 10.3390/healthcare8020088, PMID: 32272608 PMC7349118

[3] Anjel Vahratian J-MSL Bertolli J Unger ER . Myalgic encephalomyelitis/chronic fatigue syndrome in adults: United States, 2021–2022. Hyattsville, Maryland, United States: National Center for Health Statistics, Centers for Disease Control and Prevention (2023).

[4] Stahl D Rimes KA Chalder T . Mechanisms of change underlying the efficacy of cognitive behaviour therapy for chronic fatigue syndrome in a specialist clinic: a mediation analysis. Psychol Med. (2014) 44:1331–44. doi: 10.1017/S0033291713002006, PMID: 23931831

[5] Yancey JR Thomas SM . Chronic fatigue syndrome: diagnosis and treatment. Am Fam Physician. (2012) 86:741–6., PMID: 23062157

[6] Vink M Vink-Niese A . Cognitive behavioural therapy for myalgic encephalomyelitis/chronic fatigue syndrome is not effective. Re-analysis of a Cochrane review. Health Psychol Open. (2019) 6. doi: 10.1177/2055102919840614, PMID: 31080632 PMC6498783

[7] Green CR Cowan P Elk R O'Neil KM Rasmussen AL . National institutes of health pathways to prevention workshop: advancing the research on myalgic encephalomyelitis/chronic fatigue syndrome. Ann Intern Med. (2015) 162:860–5. doi: 10.7326/M15-0338, PMID: 26075757

[8] Smith MEB NH Haney E Pappas M Daeges M Wasson N McDonagh M . Diagnosis and treatment of myalgic encephalomyelitis/chronic fatigue syndrome. Agency for healthcare research and quality. In: Addendum july 2016;Evidence report/technology assessment no. 219. Rockville, Maryland, USA: Pacific Northwest Evidence-based Practice Center under Contract No. 290-2012-00014-I (2014). p. 15–E001-EF. 10.23970/AHRQEPCERTA219 30313001

[9] Price JR Mitchell E Tidy E Hunot V . Cognitive behaviour therapy for chronic fatigue syndrome in adults. Cochrane Database Syst Rev. (2008) 2008:Cd001027. doi: 10.1002/14651858.CD001027.pub2, PMID: 18646067 PMC7028002

[10] Malouff JA Thorsteinsson EB Rooke SE Bhullar N Schutte NS . Efficacy of cognitive behavioral therapy for chronic fatigue syndrome: A meta-analysis. Clin Psychol Review. (2008) 28:736–45. doi: 10.1016/j.cpr.2007.10.004, PMID: 18060672

[11] Castell BD Kazantzis N Moss-Morris RE . Cognitive behavioral therapy and graded exercise for chronic fatigue syndrome: A meta-analysis. Clin Psychology: Sci Practice. (2011) 18:311–24. doi: 10.1111/j.1468-2850.2011.01262.x

[12] Kim DY Lee JS Park SY Kim SJ Son CG . Systematic review of randomized controlled trials for chronic fatigue syndrome/myalgic encephalomyelitis (CFS/ME). J Trans Med. (2020) 18. doi: 10.1186/s12967-019-02196-9, PMID: 31906979 PMC6943902

[13] Kuut TA Buffart LM Braamse AMJ Csorba I Bleijenberg G Nieuwkerk P . Does the effect of cognitive behavior therapy for chronic fatigue syndrome (ME/CFS) vary by patient characteristics? A systematic review and individual patient data meta-analysis. psychol Med. (2024) 54:447–56. doi: 10.1017/S0033291723003148, PMID: 37927223

[14] Maas genannt Bermpohl F Kucharczyk-Bodenburg A-C Martin A . Efficacy and acceptance of cognitive behavioral therapy in adults with chronic fatigue syndrome: A meta-analysis. Int J Behav Med. (2024) 31:895–910. doi: 10.1007/s12529-023-10254-2, PMID: 38228869 PMC11588766

[15] Gotaas ME Stiles TC Bjørngaard JH Borchgrevink PC Fors EA . Cognitive behavioral therapy improves physical function and fatigue in mild and moderate chronic fatigue syndrome: A consecutive randomized controlled trial of standard and short interventions. Front Psychiatry. (2021) 12:580924. doi: 10.3389/fpsyt.2021.580924, PMID: 33912079 PMC8071989

[16] van der Schaaf ME Geerligs L Toni I Knoop H Oosterman JM . Disentangling pain and fatigue in chronic fatigue syndrome: a resting state connectivity study before and after cognitive behavioral therapy. Psychol Med. (2024) 54:1735–48. doi: 10.1017/S0033291723003690, PMID: 38193344

[17] Strand EB Mengshoel AM Sandvik L Helland IB Abraham S Nes LS . Pain is associated with reduced quality of life and functional status in patients with Myalgic Encephalomyelitis/Chronic Fatigue Syndrome. Scandinavian J Pain. (2019) 19:61–72. doi: 10.1515/sjpain-2018-0095, PMID: 30325738

[18] Page MJ McKenzie JE Bossuyt PM Boutron I Hoffmann TC Mulrow CD . The PRISMA 2020 statement: an updated guideline for reporting systematic reviews. . BMJ. (2021) 372:n71. doi: 10.1136/bmj.n71, PMID: 33782057 PMC8005924

[19] Collin SM Nuevo R van de Putte EM Nijhof SL Crawley E . Chronic fatigue syndrome (CFS) or myalgic encephalomyelitis (ME) is different in children compared to in adults: a study of UK and Dutch clinical cohorts. BMJ Open. (2015) 5:e008830. doi: 10.1136/bmjopen-2015-008830, PMID: 26510728 PMC4636651

[20] IBM . IBM SPSS statistics for windows. 29.0.2.0 ed. Armonk, NY: IBM Corp (2023).

[21] Higgins JP Thompson SG Deeks JJ Altman DG . Measuring inconsistency in meta-analyses. Bmj. (2003) 327:557–60. doi: 10.1136/bmj.327.7414.557, PMID: 12958120 PMC192859

[22] DerSimonian R Laird N . Meta-analysis in clinical trials. Control Clin Trials. (1986) 7:177–88. doi: 10.1016/0197-2456(86)90046-2, PMID: 3802833

[23] Wiborg JF van Bussel J van Dijk A Bleijenberg G Knoop H . Randomised controlled trial of cognitive behaviour therapy delivered in groups of patients with chronic fatigue syndrome. Psychother Psychosom. (2015) 84:368–76. doi: 10.1159/000438867, PMID: 26402868

[24] Núñez M Fernández-Solà J Nuñez E Fernández-Huerta JM Godás-Sieso T Gomez-Gil E . Health-related quality of life in patients with chronic fatigue syndrome: group cognitive behavioural therapy and graded exercise versus usual treatment. A randomised controlled trial with 1 year of follow-up. Clin Rheumatol. (2011) 30:381–9. doi: 10.1007/s10067-010-1475-6, PMID: 21234629

[25] Knoop H Stulemeijer M de Jong L Fiselier TJW Bleijenberg G . Efficacy of cognitive behavioral therapy for adolescents with chronic fatigue syndrome: Long-term follow-up of a randomized, controlled trial. Pediatrics. (2008) 121:E619–E25. doi: 10.1542/peds.2007-1488, PMID: 18310181

[26] Jason LA Torres-Harding S Friedberg F Corradi K Njoku MG Donalek J . Non-pharmacologic interventions for CFS: A randomized trial. J Clin Psychol Med Settings. (2007) 14:275–96. doi: 10.1007/s10880-007-9088-8

[27] O'Dowd H Gladwell P Rogers CA Hollinghurst S Gregory A . Cognitive behavioural therapy in chronic fatigue syndrome: a randomised controlled trial of an outpatient group programme. Health Technol Assess. (2006) 10:iii–iv, ix-x, 1-121. doi: 10.3310/hta10370, PMID: 17014748

[28] Deale A . Treating chronic fatigue syndrome with cognitive behaviour therapy. Ment Health Care. (1997) 1:134–7.

[29] Janse A Worm-Smeitink M Bleijenberg G Donders R Knoop H . Efficacy of web-based cognitive-behavioural therapy for chronic fatigue syndrome: Randomised controlled trial. Br J Psychiatry. (2018) 212:112–8. doi: 10.1192/bjp.2017.22, PMID: 29436329

[30] Tummers M Knoop H van Dam A Bleijenberg G . Implementing a minimal intervention for chronic fatigue syndrome in a mental health centre: A randomized controlled trial. psychol Med. (2012) 42:2205–15. doi: 10.1017/S0033291712000232, PMID: 22354999

[31] Lopez C Antoni M Penedo F Weiss D Cruess S Segotas M-C . A pilot study of cognitive behavioral stress management effects on stress, quality of life, and symptoms in persons with chronic fatigue syndrome. J Psychosomatic Res. (2011) 70:328–34. doi: 10.1016/j.jpsychores.2010.11.010, PMID: 21414452 PMC3073706

[32] Prins JB Bleijenberg G Bazelmans E Elving LD de Boo TM Severens JL . Cognitive behaviour therapy for chronic fatigue syndrome: a multicentre randomised controlled trial. Lancet. (2001) 357:841–7. doi: 10.1016/S0140-6736(00)04198-2, PMID: 11265953

[33] Knoop H van der Meer JW Bleijenberg G . Guided self-instructions for people with chronic fatigue syndrome: randomised controlled trial. Br J Psychiatry. (2008) 193:340–1. doi: 10.1192/bjp.bp.108.051292, PMID: 18827302

[34] Burgess M Andiappan M Chalder T . Cognitive behaviour therapy for chronic fatigue syndrome in adults: face to face versus telephone treatment: a randomized controlled trial. Behav Cognit Psychother. (2012) 40:175–91. doi: 10.1017/S1352465811000543, PMID: 21929831

[35] Vos-Vromans D Evers S Huijnen I Köke A Hitters M Rijnders N . Economic evaluation of multidisciplinary rehabilitation treatment versus cognitive behavioural therapy for patients with chronic fatigue syndrome: A randomized controlled trial. PloS One. (2017) 12:e0177260. doi: 10.1371/journal.pone.0177260, PMID: 28574985 PMC5456034

[36] Söderberg S Evengård B . Short-term group therapy for patients with chronic fatigue syndrome. Psychother Psychosom. (2001) 70:108–11. doi: 10.1159/000056234, PMID: 11244392

[37] Hall DL Lattie EG Milrad SF Czaja S Fletcher MA Klimas N . Telephone-administered versus live group cognitive behavioral stress management for adults with CFS. J Psychosom Res. (2017) 93:41–7. doi: 10.1016/j.jpsychores.2016.12.004, PMID: 28107891 PMC5270375

[38] Wadaa NN . The effectiveness of cognitive behavior therapy to reduce chronic fatigue syndrome among Iraqi employees. Dirasat: Hum Soc Sci. (2020) 47:425–33. doi: 10.35516/0103-047-002-028

[39] Lattie EG . The effects of telephone-delivered cognitive behavioral stress management on inflammation and symptoms in Myalgic Encephalomyelitis/Chronic Fatigue Syndrome: A computational immunology approach [Ph.D.]. Coral Gables, Florida, USA: University of Miami (2016).

[40] Borenstein MH Higgins J Rothstein H . Subgroup analyses. In: Introduction to meta-analysis. Chichester, West Sussex, United Kingdom: John Wiley & Sons, Ltd.; (2009). p. 149–86.

[41] Brurberg KG Fønhus MS Larun L Flottorp S Malterud K . Case definitions for chronic fatigue syndrome/myalgic encephalomyelitis (CFS/ME): a systematic review. BMJ Open. (2014) 4:e003973. doi: 10.1136/bmjopen-2013-003973, PMID: 24508851 PMC3918975

[42] Enander J Ljótsson B Anderhell L Runeborg M Flygare O Cottman O . Long-term outcome of therapist-guided internet-based cognitive behavioural therapy for body dysmorphic disorder (BDD-NET): a naturalistic 2-year follow-up after a randomised controlled trial. BMJ Open. (2019) 9:e024307. doi: 10.1136/bmjopen-2018-024307, PMID: 30647044 PMC6340432

[43] Andersson E Steneby S Karlsson K Ljótsson B Hedman E Enander J . Long-term efficacy of Internet-based cognitive behavior therapy for obsessive–compulsive disorder with or without booster: a randomized controlled trial. psychol Med. (2014) 44:2877–87. doi: 10.1017/S0033291714000543, PMID: 25066102

[44] Philips B Falkenström F . What research evidence is valid for psychotherapy research? Front Psychiatry. (2020) 11:625380. doi: 10.3389/fpsyt.2020.625380, PMID: 33505325 PMC7829194

[45] Cella M White PD Sharpe M Chalder T . Cognitions, behaviours and co-morbid psychiatric diagnoses in patients with chronic fatigue syndrome. psychol Med. (2013) 43:375–80. doi: 10.1017/S0033291712000979, PMID: 22571806

[46] Natelson BH Lin J-MS Lange G Khan S Stegner A Unger ER . The effect of comorbid medical and psychiatric diagnoses on chronic fatigue syndrome. Ann Med. (2019) 51:371–8. doi: 10.1080/07853890.2019.1683601, PMID: 31642345 PMC7877877

[47] Vîslă A Constantino M Flückiger C . Predictors of change in patient treatment outcome expectation during cognitive-behavioral psychotherapy for generalized anxiety disorder. Psychother Theory Res Practice. (2021) 58:219–29. doi: 10.1037/pst0000371, PMID: 34410791

[48] Fernandez E Salem D Swift JK Ramtahal N . Meta-analysis of dropout from cognitive behavioral therapy: Magnitude, timing, and moderators. J Consult Clin Psychol. (2015) 83:1108–22. doi: 10.1037/ccp0000044, PMID: 26302248

[49] Bados A Balaguer G Saldaña C . The efficacy of cognitive-behavioral therapy and the problem of drop-out. J Clin Psychol. (2007) 63:585–92. doi: 10.1002/jclp.20368, PMID: 17457848

[50] Westmacott R Hunsley J . Reasons for terminating psychotherapy: a general population study. J Clin Psychol. (2010) 66:965–77. doi: 10.1002/jclp.20702, PMID: 20694960

[51] Sharf J Primavera LH Diener MJ . Dropout and therapeutic alliance: a meta-analysis of adult individual psychotherapy. Psychother (Chic). (2010) 47:637–45. doi: 10.1037/a0021175, PMID: 21198249

[52] Swift JK Greenberg RP . Premature discontinuation in adult psychotherapy: a meta-analysis. J Consult Clin Psychol. (2012) 80:547–59. doi: 10.1037/a0028226, PMID: 22506792

[53] Cuijpers P . Targets and outcomes of psychotherapies for mental disorders: an overview. World Psychiatry. (2019) 18:276–85. doi: 10.1002/wps.20661, PMID: 31496102 PMC6732705

[54] Mao J . Challenges of managing chronic pain. BMJ. (2017) 356:j741. doi: 10.1136/bmj.j741, PMID: 28213344

[55] Cella DF . Quality of life: Concepts and definition. J Pain Symptom Management. (1994) 9:186–92. doi: 10.1016/0885-3924(94)90129-5, PMID: 8014530

[56] Worm-Smeitink M Nikolaus S Goldsmith K Wiborg J Ali S Knoop H . Cognitive behaviour therapy for chronic fatigue syndrome: Differences in treatment outcome between a tertiary treatment centre in the United Kingdom and the Netherlands. J Psychosomatic Res. (2016) 87:43–9. doi: 10.1016/j.jpsychores.2016.06.006, PMID: 27411751

[57] Chalder T Deary V Husain K Walwyn R . Family-focused cognitive behaviour therapy versus psycho-education for chronic fatigue syndrome in 11- to 18-year-olds: a randomized controlled treatment trial. . psychol Med. (2009) 40:1269–79. doi: 10.1017/S003329170900605X, PMID: 19891804

[58] Twisk FN Maes M . A review on cognitive behavorial therapy (CBT) and graded exercise therapy (GET) in myalgic encephalomyelitis (ME)/chronic fatigue syndrome (CFS): CBT/GET is not only ineffective and not evidence-based, but also potentially harmful for many patients with ME/CFS. Neuro Endocrinol Lett. (2009) 30:284–99., PMID: 19855350

[59] Deary V Chalder T Sharpe M . The cognitive behavioural model of medically unexplained symptoms: a theoretical and empirical review. Clin Psychol Rev. (2007) 27:781–97. doi: 10.1016/j.cpr.2007.07.002, PMID: 17822818

[60] Chalder T . CBT for chronic fatigue syndrome London. United Kingdom: King's College London (2014). Available online at: https://www.kcl.ac.uk/news/spotlight/cbt-for-chronic-fatigue-syndrome.

[61] Nacul L Authier FJ Scheibenbogen C Lorusso L Helland IB Martin JA . European network on myalgic encephalomyelitis/chronic fatigue syndrome (EUROMENE): expert consensus on the diagnosis, service provision, and care of people with ME/CFS in europe. Med (Kaunas). (2021) 57. doi: 10.3390/medicina57050510, PMID: 34069603 PMC8161074

[62] Bateman L Bested AC Bonilla HF Chheda BV Chu L Curtin JM . Myalgic encephalomyelitis/chronic fatigue syndrome: essentials of diagnosis and management. Mayo Clin Proc. (2021) 96:2861–78. doi: 10.1016/j.mayocp.2021.07.004, PMID: 34454716

