# Supplementary material for: Cognitive behavioural therapy for the treatment of chronic fatigue syndrome in adults – a meta-analysis
Source: Front Psychiatry. 2025 Oct 31;16:1647897. doi: 10.3389/fpsyt.2025.1647897 (PMC12616423; doi:10.3389/fpsyt.2025.1647897)
Supplement: Supplementary file 1 [file SupplementaryFile1.docx]

Appendix 1: Study intervention and comparison details

|  | **Intervention** | **Intervention details** | **Additional interventions** | **Comparator** | **Comparator details** | **Reported primary outcomes** | **Adverse events** | **Dropouts** |
| --- | --- | --- | --- | --- | --- | --- | --- | --- |
| van der Schaaf et al. (16) (n=51) | Individual face-to-face CBT (n=33) | 12-14 sessions over 6 months | Grading of physical activities as per CBT protocol | Waiting list (n=18) | Did not receive any treatment or control intervention, and started CBT after second assessment | Fatigue severity and neuroimaging data collection | Nil reported | 18.1% in intervention group and 4.5% in comparator group |
| Gotaas et al. (15) (n=236) | Individual face-to-face CBT (n=156) | Arm 1: 16 x individual-face-to-face CBT over 4 months  Arm 2: 8 weekly sessions of interpersonal CBT over 2 months | Graded activity based on fear avoidance model | Waiting list (n=80) | Nil intervention for 16 weeks from baseline, then offered interpersonal CBT | Physical function | 2% in intervention group, and 5% in comparator reported increased fatigue, nausea, headache, pain in joints, but no serious adverse events | 13.5% in intervention group and 10% in comparator group |
| Janse et al. (29) (n=240) | Self-directed CBT (n=160) | 7 self-directed CBT modules over 6 months.  Arm 1: therapist offered feedback at specific timepoints Arm 2: where feedback was provided when asked by patient | 25 patients started another intervention during study | Waiting list (n=80) | Waiting period for 6 months | Fatigue severity | 11% patients in the protocol-driven condition, 18% in the on-demand condition, and 26% in the control condition | 7.5% from intervention group and 5% from comparator group |
| Wiborg et al. (23) (n=204) | Group CBT (n=136) | 14 x 2 hour group sessions over 6 months | Exercise therapy not part of intervention | Waiting list (n=68) | Did not specify | Fatigue severity | Nil reported | 43.4% from intervention group and 14.7% from comparator group |
| Tummers et al. (30) (n=123) | Self-directed CBT (n=62) | Self-directed CBT via booklet which contained information about CFS, alongside assignments over 5 months | Suggestions for graded activity discussed | Waiting list | Did not specify | Fatigue severity | Nil reported | 11.2% from intervention group and 8.2% from comparator group |
| Núñez et al. (24) (n=120) | Group CBT (n=60) | Fortnightly 90 minute group sessions in groups of 16 for 3 months, plus GET and symptomatic pharmacology | Graded exercise | Treatment as usual (n=60) | Exercise counselling program which included 2 x 10 minute daily exercises. Symptomatic pharmacology as per intervention group | Quality of life | Intervention group reported deterioration in physical functioning and pain | 3.3% from intervention group and 5% from comparator group |
| Lopez et al. (31) (n=69) | Group CBT (n=44) | 12 x 2 hour weekly group meetings over 3 months | Nil information available | Psychoeducational seminar control (n=25) | Half day psychoeducational seminar led by a post-doctoral clinical fellow and advanced psychology graduate students | Stress, quality of life, and CFS symptoms | Nil reported | 13.6% from intervention group and 20% from comparator group |
| Knoop et al. (33) (n=169) | Self-directed CBT (n=84) | Self-directed instruction booklet aimed to decrease focus on bodily symptoms over 4 months | Structured physical activity program | Usual care (n=85) | Waiting list | Fatigue severity | Nil reported | 19% from intervention group and 4.7% from comparator group |
| Jason et al. (26) (n=114) | Individual face-to-face CBT (n=29) | 13 x 45 minute fortnightly individual face-to-face sessions over 6 months | Schedule of planned, graded activity | Cognitive therapy + anaerobic therapy + relaxation (n=85) | Cognitive therapy:  developing cognitive  strategies to better tolerate and reduce stress and symptoms  Anaerobic therapy: developing individualised,  constructive and pleasurable activities accompanied by  reinforcement of progress  Relaxation: several types of relaxation demonstrated | CFS symptoms and physical functioning | Comparator group experienced negative change for pain severity, tender lymph nodes, pain in joints and impaired memory | 25% dropout rate in both intervention and comparator groups |
| O’Dowd et al. (27) (n=153) | Group CBT (n=52) | 8 x 2 hour fortnightly group sessions in groups of 8-12 given over 4 months | Graded exercise therapy | Education and support group + standard medical treatment (n=101) | Education and support group: sharing of experiences and learning of basic relaxation  Standard medical treatment: did not attend hospital other than to complete assessment skills | Physical functioning and mental functioning | Nil reported | 20% dropout rate in intervention group, 10.9% in comparator group |
| Prins et al. (32) (n=278) | Individual face-to-face CBT (n=93) | 16 x 1 hour face-to-face individual CBT sessions over 8 months | Structured activity regime. Patients free to have other examinations or treatments | Support group + natural course (n=185) | Support group: 11 x 1.5hr meetings over 8 months. Natural course: no interventions offered, patients free to have other treatments | Fatigue severity and functional impairment | Nil reported | 40.9% dropout rate in intervention group and 29.2% in comparator group |
| Deale et al. (28) (n=60) | Individual face-to-face CBT (n=30) | 13 x individual face-to-face CBT weekly or fortnightly sessions over 4-6 months | Scheduled, graded activity, reported one patient started antidepressant, and one patient visited homeopath | Relaxation (n=30) | 13 sessions involving progressive muscle relaxation, visualisation and rapid relaxation (n=30) | Functional impairment, fatigue, psychological distress, mood | Nil reported | 10% dropout rate in intervention group and 13% in comparator group |
|  |  | | | | | |  |  |
